# A prediction model of cognitive impairment risk in elderly illiterate Chinese women

**DOI:** 10.3389/fnagi.2023.1148071

**Published:** 2023-04-26

**Authors:** Zhaojing Chen, Jiaolan Du, Qin Song, Jun Yang, Yinyin Wu

**Affiliations:** ^1^Department of Epidemiology and Health Statistics, School of Public Health, Hangzhou Normal University, Hangzhou, China; ^2^Department of Occupational and Environmental Health, School of Public Health, Hangzhou Normal University, Hangzhou, China; ^3^Department of Nutrition and Toxicology, School of Public Health, Hangzhou Normal University, Hangzhou, China

**Keywords:** cognitive impairment, risk factors, prediction model, elderly, illiterate, nomogram

## Abstract

**Objective:**

To establish and validate a targeted model for the prediction of cognitive impairment in elderly illiterate Chinese women.

**Methods:**

1864 participants in the 2011–2014 cohort and 1,060 participants in the 2014–2018 cohort from the Chinese Longitudinal Healthy Longevity Survey (CLHLS) were included in this study. The Chinese version of the Mini-Mental State Examination (MMSE) was used to measure cognitive function. Demographics and lifestyle information were collected to construct a risk prediction model by a restricted cubic spline Cox regression. The discrimination and accuracy of the model were assessed by the area under the curve (AUC) and the concordance index, respectively.

**Results:**

A total of seven critical variables were included in the final prediction model for cognitive impairment risk, including age, MMSE score, waist-to-height ratio (WHtR), psychological score, activities of daily living (ADL), instrumental abilities of daily living (IADL), and frequency of tooth brushing. The internal and external validation AUCs were 0.8 and 0.74, respectively; and the receiver operating characteristic (ROC) curves indicated good performance ability of the constructed model.

**Conclusion:**

A feasible model to explore the factors influencing cognitive impairment in elderly illiterate women in China and to identify the elders at high risk was successfully constructed.

## Introduction

Currently, population aging is becoming a major social issue to almost all countries over the world. There are many public health concerns associated with population aging, and dementia is among the most significant ones. According to the World Health Organization estimation, about 50 million elderly people are suffering from dementia. Among them, China has the largest number of dementia patients with nearly 25% of the world’s dementia cases, and an annual increase of more than 0.36 million cases (Jiang et al., 2020). Dementia decreases the quality of life of patients, and has imposed a heavy burden on social and medical resources. Unfortunately, there are still no effective treatments for dementia. Therefore, it is crucial to identify cognitive impairment at an early stage to prevent or delay its progression to dementia in elderly population.

Many studies have explored the risk factors as well as constructed various prediction models for cognitive impairment. Among the factors that influence cognitive function, gender and education have been identified as important ones in elderly adults. For example, several studies have shown that there are significant differences in the risk of developing cognitive impairment between men and women, with elderly women at a higher risk of cognitive impairment (Gavrila et al., 2009; Lei and Chen, 2020; Ni et al., 2020). Besides, low educational attainment is one of the main non-genetic risk factors for dementia due to inadequate cognitive reserve (Qiu et al., 2005; Kaup et al., 2014). Thus, we expect elderly illiterate women to be among the populations with one of the highest risk for cognitive impairment. For prediction models, Byeon (2015) had developed a prediction model for mild cognitive impairment with predictors including demographic characteristics and diseases; and Suyeong et al. (2022) used the 2018 Korean Longitudinal Study of Aging to develop a cognitive impairment prediction model for community-dwelling older adults. However, those models still suffered certain limitations. For instance, most studies had insufficient sample sizes (Schrag et al., 2017; Zhang et al., 2022), or had participants from developed countries (Barnes et al., 2014). Other studies lacked validation (Pankratz et al., 2015), or used specific type of patients as participants, such as those suffering from diabetes (Samoilova et al., 2020). In addition, there is no prognostic model for cognitive impairment risk specifically for elderly illiterate women.

Therefore, in the current study, we intended to establish a prediction model for the risk of cognitive impairment among elderly illiterate women in China, using data from the largest national cohort study in China, the Chinese Longitudinal Healthy Longevity Survey (CLHLS) [Chinese Longitudinal Healthy Longevity Survey (CLHLS), 2021].

## Materials and methods

### Data source

CLHLS was the first longitudinal survey on the determinants of healthy aging in China. This survey was conducted face-to-face using an internationally compatible questionnaire in 23 provinces covering 85% of the total population of China. CLHLS conducted a multi-stage sampling to ensure participants were reasonably dispersed (Shen and Zeng, 2014). The baseline survey was conducted in 1998, and the follow-up surveys were completed in year 2000, 2002, 2005, 2008–2009, 2011–2012, 2014, and 2018.

### Study participants

Participants were included if they were (1) aged 60 years or above, (2) females who were confirmed as illiterate, (3) normal cognitive function at baseline survey, and (4) had given consent to participate in this study. Participants with serious physical illnesses, who died or were lost during the follow-up were excluded. Finally, 1864 eligible participants from the 2011–2014 CLHLS cohort were screened for the construction of the prediction model, with 70% of the sample size as the training set and 30% as the test set. Following the same criteria, 1,060 eligible participants from the 2014–2018 CLHLS cohort were selected for external validation of the prediction model. A detailed flow chart of participant selection was shown in Supplementary Figure S1.

### Assessment of cognitive impairment

Cognitive function was measured by the Chinese version of Mini-Mental State Examination (MMSE), which is widely used for cognitive screening and has been proven to be a simple test for memory, orientation, verbal computation, and attention (Folstein et al., 1975). It has good applicability in China (Cui et al., 2011), especially for elderly people who cannot undergo complex clinical diagnostic tests (Patrizio et al., 2008). There are 24 questions on the scale and the score of MMSE ranges from 0 to 30 points, with lower scores indicating worse cognitive function (Li et al., 2018). In our study, an MMSE score of <18 was defined as cognitive impairment (Cui et al., 2011). Cognitive function was assessed every 3 years, and the date of cognitive impairment was recorded as endpoint time.

### Candidate predictors

The selection of variables was based on scientific knowledge, clinical importance, and predictors identified in previously published studies, including demographics, lifestyle, physical function, mental health, leisure activities, dietary habits, anthropometric index, and chronic diseases history.

Continuous variables included were age, body mass index (BMI), blood pressure, ability to perform activities of daily living (ADL), instrumental abilities of daily living (IADL) scores, baseline MMSE scores, psychological scores, sleep duration, nearest medical facility distance, and alimony paid by children. ADL was assessed based on six items and the score ranged from 6 to 18. IADL was evaluated based on eight items and the score ranged from 8 to 24 (Yang et al., 2014). Higher scores indicated weaker physical function. As BMI cannot be used independently to determine body fat distribution in the elderly population, so we added the waist-to-height ratio (WHtR) as a health-related supplementary index. For women, 0.49–0.57 was considered overweight, and above 0.57 was considered centrally obese (Sun et al., 2020).

Other variables were classified as follows: gender was defined as male or female; marital status was defined as living without a spouse or living with a spouse; occupation was defined as desk job, manual work, or no fixed occupation; the residence was defined as urban or rural; the main cooking fuel in the household was defined as clean fuel (electricity, natural gas, solar) or polluting fuel (kerosene, charcoal, firewood, coal); the economic situation was classified as rich, average, or poor according to the self-assessment; the intake frequencies of vegetables, meat, fish, and eggs were recorded as “almost every day,” “occasionally,” or “rarely or never”; self-reported frequency of leisure activities was defined as never, occasional, and often. Interactions between age and other variables were also taken into account.

### Statistical analysis

Model construction and validation were performed using the complete datasets, with the 2011–2014 cohort as the development cohort and the 2014–2018 cohort as the validation cohort. All variables with missing rates higher than 10% were excluded from this study and remaining missing variables were imputed by using median and mode. Baseline characteristics were presented using mean ± standard deviation (SD) for continuous-type variables while counts and percentages were used for categorical variables.

In recent years, machine learning algorithms have been increasingly used to build disease prediction models. Random survival forest (RSF), as a type of machine learning, has shown excellent efficacy in several studies (Ishwaran et al., 2008). Our study involved survival data for a large number of variables, which was more suitable to use the RSF algorithm. RSF takes time into account and is used specifically for the analysis of survival data in prospective cohorts. However, machine learning algorithms also have some drawbacks. For example, it is difficult to be applied in the clinical field, because the machine learning model contains a large number of variables that are hard to explain (Shin et al., 2020). Therefore, we used the RSF algorithm for variable screening and then added Cox regression to construct the model, which can maximize model efficacy and reduce model complexity.

First of all, we used RSF for variable screening, which can evaluate the importance of each variable in constructing the model (Hemant et al., 2014). The main metrics of importance scoring were variable importance (VIMP) and minimum depth (MD). The combination of these two metrics can effectively prevent overfitting. Thus, they were both used for variable screening (Chen et al., 2021). RSF was conducted twice to screen the variables correlated with cognitive impairment for the follow-up study. The first RSF was conducted based on the whole raw data of the development cohort for noise reduction and variables screening; the second one was conducted using the training set of the development cohort for variable screening.

And then, the variables obtained by the above screening were subjected to univariate and multivariate Cox regression analysis to explore the independent risk factors, and to construct the cognitive impairment prediction. Multivariable time-to-event analysis was performed using Cox proportional hazards regression model. The estimated risk for the variables was presented as hazard ratio (HR).

It is necessary to examine whether the survival data satisfied the proportional hazard (PH) assumption before applying the Cox regression model. Test methods include graphical (Hess, 1995) and hypothesis test (Nicholas, 1997). Schoenfeld residual plots can be used to visually judge whether the PH assumption holds by looking at the distribution of scatter points in the scatter plot. However, it is difficult to determine the extent of deviation from the PH assumption. So, we added the cubic spline function method to test the PH assumption further. The cubic spline function is a flexible non-parametric method for testing PH assumption in survival analysis. The function estimates the hazard ratio over time and compares it to a constant hazard ratio using a statistical test (such as a likelihood ratio test or a score test). If the test indicates that the hazard ratio is not constant over time, then the PH assumption is violated (Patrick and Mahesh, 2002). It is inappropriate to continue using the traditional Cox model if survival data do not meet the PH assumption. The curvilinear relationships can be fitted in the Cox model framework by restricted cubic splines (RCS) which estimates the effects of variables over time (Dong, 2011).

The nomogram can show the results of the prediction model in a visual way. A score is assigned to each predictor variable according to the magnitude of the regression coefficient, and the predicted value of cognitive impairment in that individual can be calculated easily (Hu et al., 2019).

The accuracy and discrimination performances of the model were quantitatively assessed by the concordance index (C-index) and the area under curve (AUC) of the receiver operating characteristic (ROC) curve. In general, C-index in the range of 0.5–0.7 indicates a low accuracy; in the range of 0.7–0.9 indicates a moderate accuracy; and above 0.9 indicates a high accuracy (Harrell et al., 1996). The AUC value ranges from 0.5 to 1, with value 1 being the best discriminatory ability. The test set was used for internal validation and the 2014–2018 cohort was used for external validation to evaluate the general applicability of the model. The schematic diagram of the technical route was shown in Supplementary Figure S2.

All statistical analyses were conducted using IBM SPSS 22.0 and R 4.1.0 (R Core Team, 2021). The main packages used were “foreign,” “mice,” “nnet,” “DataExplorer,” “dplyr,” “rpart,” “akima,” “ggplot2,” “randomForest,” “randomForestSRC,” “ggRandomForests,” “rms,” “tidyverse,” “MASS,” “survival,” “survminer” and “ROCR” packages. The test level was bilateral, and *p* < 0.05 was considered statistically significant.

## Results

### Baseline characteristics

Table 1 showed the baseline characteristics of the development and validation cohorts. There were 1864 elderly illiterate women with an average age of 83.7 years (SD = 9.87) in the development cohort. The age ranged from 62 to 112 and the median follow-up time was 2.76 (IQR: 2.27–2.86) years. The mean baseline MMSE was 26.11 ± 3.47 and the mean IADL score was 12.17 ± 4.9; the mean psychological score was 18.24 ± 3.61; the mean WHtR was 0.54 ± 0.08. There were 1,060 elderly illiterate women with an average age of 83.01 years (SD = 8.51) in the external validation cohort. Table 2 showed the baseline characteristics of the participants between the training set and test set in the development cohort. There was no significant difference in the distribution of basic characteristics between the training and test sets.

**Table 1:** Baseline characteristics of the participants in the development and validation cohorts.

Variables	Development cohort (*n* = 1864)	Validation cohort (*n* = 1,060)
Age (mean ± SD, years)	83.70 ± 9.87	83.01 ± 8.51
*Age group (frequency (%))*		
60–69	100 (5.36)	30 (2.83)
70–79	593 (31.81)	361 (34.06)
80–89	647 (34.71)	437 (41.23)
≥90	524 (28.11)	232 (21.89)
MMSE score (mean ± SD, points)	26.11 ± 3.47	26.09 ± 3.38
ADL score (mean ± SD, points)	6.41 ± 1.30	6.42 ± 1.32
IADL score (mean ± SD, points)	12.17 ± 4.90	11.57 ± 4.47
Psychological score (mean ± SD, points)	18.24 ± 3.61	18.03 ± 3.36
*Brushing frequency (frequency (%))*		
No brushing	847 (45.44)	412 (38.87)
Occasional brushing of teeth	206 (11.05)	127 (11.98)
Once a day	616 (33.05)	490 (46.23)
Twice a day or more	195 (10.46)	31 (2.92)
WHtR (mean ± SD)	0.54 ± 0.08	0.54 ± 0.08
Median follow-up time (IQR; years)	2.76 (2.27–2.86)	4.08 (3.51–4.30)

**Table 2:** Baseline characteristics of the participants in the training set and test set of the development cohort.

Variables	Training set (*n* = 1,304)	Test set (*n* = 560)
Age (mean ± SD, years)	83.94 **±** 10.20	83.13 **±** 9.05
*Age group (frequency (%))*		
60–69	81 (6.21)	19 (3.39)
70–79	402 (30.83)	191 (34.11)
80–89	441 (33.82)	206 (36.79)
≥90	380 (29.14)	144 (25.71)
MMSE score (mean ± SD, points)	26.16 **±** 3.46	26.00 **±** 3.49
ADL score (mean ± SD, points)	6.42 **±** 1.32	6.37 **±** 1.26
IADL score (mean ± SD, points)	12.30 **±** 5.00	11.88 **±** 4.64
Psychological score (mean ± SD, points)	18.30 **±** 3.61	18.10 **±** 3.61
*Brushing frequency (frequency (%))*		
No brushing	589 (45.17)	258 (46.07)
Occasional brushing of teeth	149 (11.43)	57 (10.18)
Once a day	420 (32.21)	196 (35.00)
Twice a day or more	146 (11.20)	49 (8.75)
WHtR (mean ± SD)	0.52 **±** 0.06	0.52 **±** 0.07
Median follow-up time (IQR; years)	2.75 (2.26–2.85)	2.77 (2.50–2.86)

### Incidence of cognitive impairment

In the development cohort, the incidence of cognitive impairment was 24.68% (*n* = 460), with an incidence density of 94.85/1000 person-years. The morbidity of cognitive impairment tended to increase with age. Especially, the incidence rate in participants aged 90 years and older was about twice than that in participants aged 80–89 years. And in the validation cohort, the incidence of cognitive impairment was 24.81% (*n* = 263), with a prevalence density of 62.24/1000 person-years. Furthermore, the incidence density of cognitive in participants aged ≥90 years was 7.3 times higher than that in participants aged 60–69 years in the development cohort. In the validation cohort, the incidence density in participants aged ≥90 years was 3.5 times higher than that in the 60–69 years age group. The detailed information can be seen in Table 3.

**Table 3:** Occurrence of cognitive impairment in different age groups in the development cohort and external validation cohort.

Group	Development cohort (*n* = 1864)				External validation cohort (*n* = 1,060)			
	Number of occurrences	Person-year of follow up	Incidence	Incidence density/1000per-year	Number of occurrences	Person-year of follow up	Incidence	Incidence density/1000per-year
*Age group (years)*								
60–69	7	260.96	7.00%	26.82	4	112.91	13.33%	35.43
70–79	53	1596.89	8.94%	33.19	43	1492.65	11.91%	28.81
80–89	143	1682.66	22.10%	84.98	107	1740.62	24.49%	61.47
≥90	257	1309.41	49.05%	196.27	109	879.64	46.98%	123.91
All	460	4849.92	24.68%	94.85	263	4225.82	24.81%	62.24

### Predictor variables of cognitive impairment

**Figure 1:**
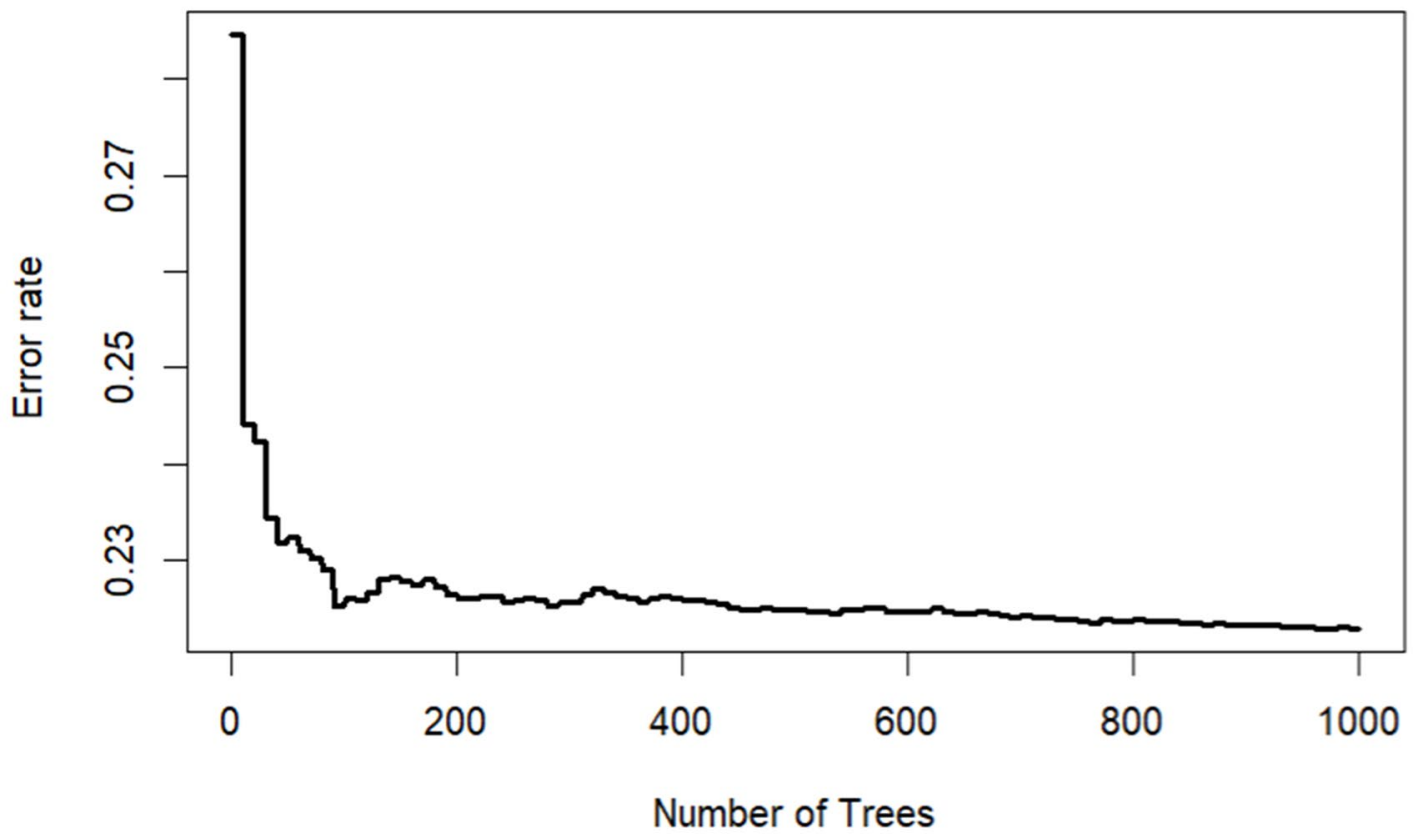
Model prediction error rate for different number of survival trees.

In this study, all variables with a missing rate of less than 10% in the 2011 baseline were used as explanatory variables. As seen in Figure 1, the random survival forest model performed stably when the number of survival trees was 500. If the number of survival trees continued to increase, the computation would have taken longer, and the model would have risked overfitting. Therefore, we constructed the dimensionality reduction model based on 500 survival tree.

The primary screening of variables can be performed by MD combined with VIMP methods. A variable whose VIMP >0 was incorporated because it improved the prediction accuracy and 46 candidate variables were included, as shown in Supplementary Table S1. The 46 variables were analyzed for covariance diagnostics, and those which had covariance with other variables were removed.

The second RSF calculation was performed in the training set of the development cohort. Supplementary Figure S3 was a scatter plot drawn by MD combined with VIMP methods. The points on the red dashed diagonal line represented the same ranking of the variables calculated by the two methods, the points above the diagonal line represented the higher ranking of importance by the VIMP method, and the points below the diagonal line represented the higher ranking of importance by the MD method. The best combination of variables was presented in Supplementary Figure S3. We chose the points under the red dashed horizontal line, namely age, IADL score, MMSE score, psychological score, number of teeth, brushing frequency, WHtR, ADL score, number of children, consumption of staple food, number of sons, and BMI.

### Model for predicting cognitive impairment

The results of univariate analysis were shown in Supplementary Table S2. The results showed that age, IADL score, MMSE score, WHtR, psychological score, BMI, brushing frequency, number of teeth, staple food consumption, and ADL score were significantly associated with the risk of cognitive impairment (*p* < 0.05).

**Figure 2:**
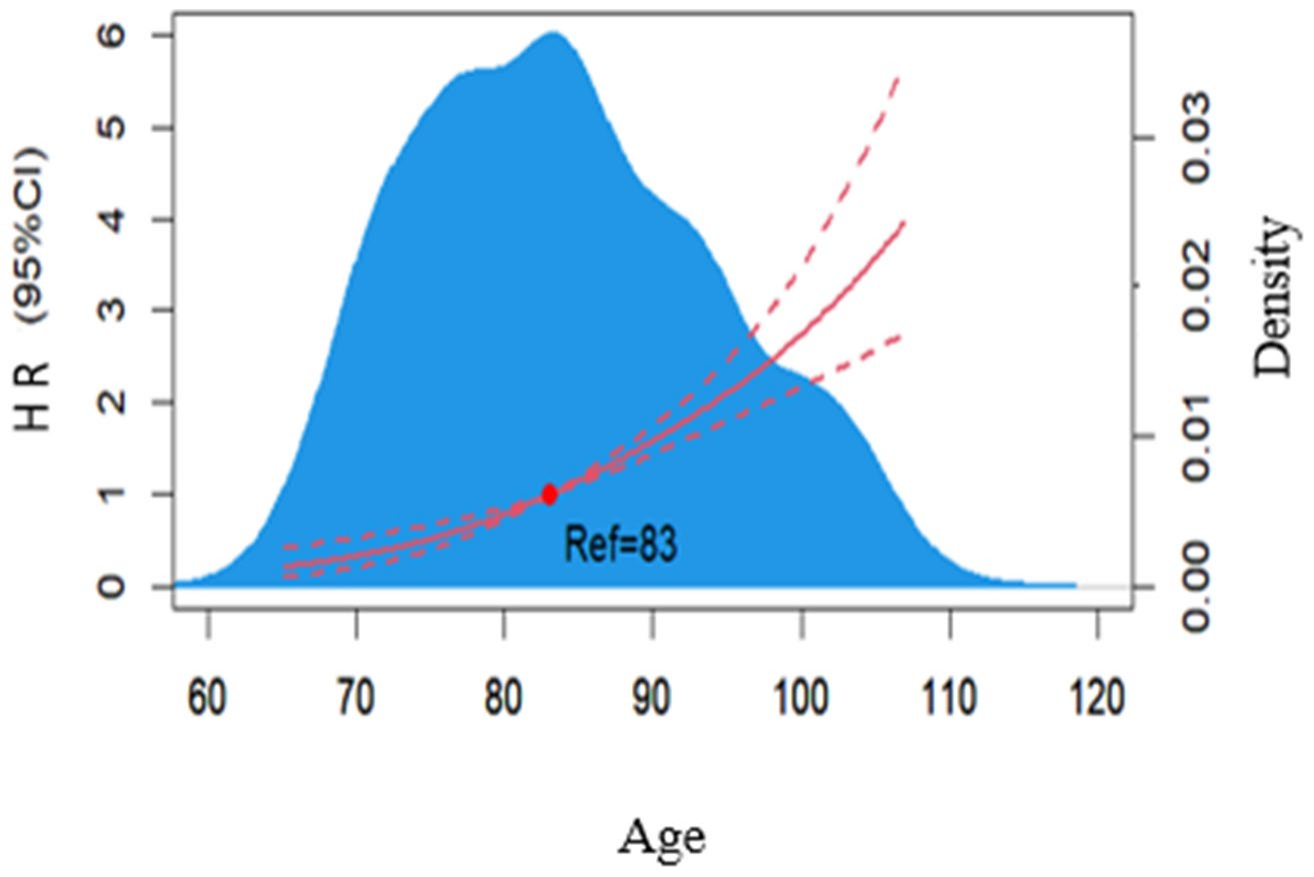
Dose–response relationship between age and risk of developing cognitive impairment in a multifactorial model. The blue area is the kernel density plot for age, and the red curve is the sample smoothing curve for age.

Then we used the multivariate Cox regression analyses to further select predictors based on the results of univariate analysis. The results of schoenfeld residual plots and RCS (Wald χ ^2^ = 5.65, *p* = 0.0174) showed that age was not consistent with the PH assumption. As shown in Figure 2, the risk of cognitive impairment increased with age after adjusting for other factors, especially when the age was ≥83 years. Thus, the Cox proportional hazard model could not be constructed directly. Finally, we constructed a RCS Cox regression model to fit the curvilinear relationship between the predictors and cognitive impairment.

As shown in Table 4, after incorporating the segmented polynomial of age into the Cox regression model and performing stepwise regression analysis, the model with the smallest AIC value was selected according to the AIC criterion (Malloy et al., 2009). Seven predictors were selected to be incorporated into the final model, including age, IADL score, ADL score, baseline MMSE score, WHtR, psychological score, and tooth brushing frequency.

**Table 4:** HR (95% CI) distribution of predictors for the final inclusion of the Cox regression model with RCS.

Predictor	Beta coefficient	S.E.	*Z*	*p*	HR (95% CI)
Age	0.088	0.021	4.156	<0.001	1.092 (1.048–1.139)
Age’	−0.028	0.022	−1.265	0.206	0.973 (0.932–1.015)
IADL Score	0.024	0.014	1.707	0.088	1.024 (0.996–1.053)
ADL Score	0.054	0.036	1.507	0.132	1.055 (0.984–1.132)
MMSE	−0.046	0.017	−2.742	0.006	0.955 (0.924–0.987)
WHtR	−2.450	0.729	−3.363	<0.001	0.086 (0.021–0.360)
Psychological score	−0.032	0.015	−2.081	0.037	0.969 (0.940–0.998)
*Brushing frequency*					
*No brushing*					
Occasional brushing	0.165	0.191	0.860	0.390	1.179 (0.810–1.715)
Once a day	−0.212	0.145	−1.465	0.143	0.809 (0.609–1.074)
Twice a day	−0.535	0.254	−2.110	0.035	0.586 (0.356–0.963)

‘Refers to that age was included in the model as an RCS cube term.

**Figure 3:**
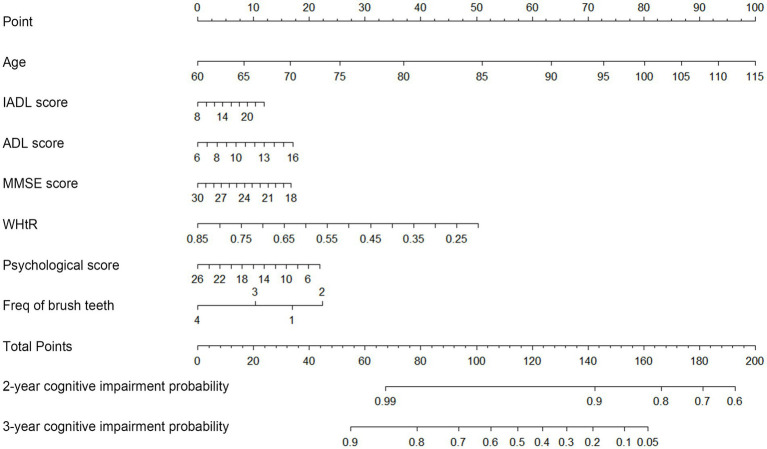
Nomogram of the Cox regression model with RCS for predicting the probability of cognitive impairment. To use the nomogram, points are produced for each factor by drawing a vertical line from the predictor’s value up to the points’ axis, and add the points from all of the factors. From the total points’ axis, a vertical line is then drawn to the 2-, and 3-year Risk axis, which yields a participant’s overall 2-, and 3-year cognitive impairment risk.

A nomogram for predicting 2-, and 3-year morbidity was constructed based on the seven significant variables in the training set. Figure 3 illustrated that age contributed most to the cognitive impairment, followed by WHtR. In the nomogram, the individual score of each factor was obtained by projecting the value of the factor vertically onto the first row named “Points.” For each participant, the total points were calculated by adding up the score of each factor. By vertically projecting total scores onto the bottom scale, we would obtain the predicted probability of occurrence of cognitive impairment. Assuming a 70-year-old illiterate female with IADL score of 14, ADL score of 10, MMSE score of 21, WhtR of 0.45, psychological score of 14, and brushing twice a day, her total score was 104 and the probability of cognitive impairment in the 2nd and 3rd year was estimated to be 5 and 37%, respectively.

### Model validation

**Figure 4:**
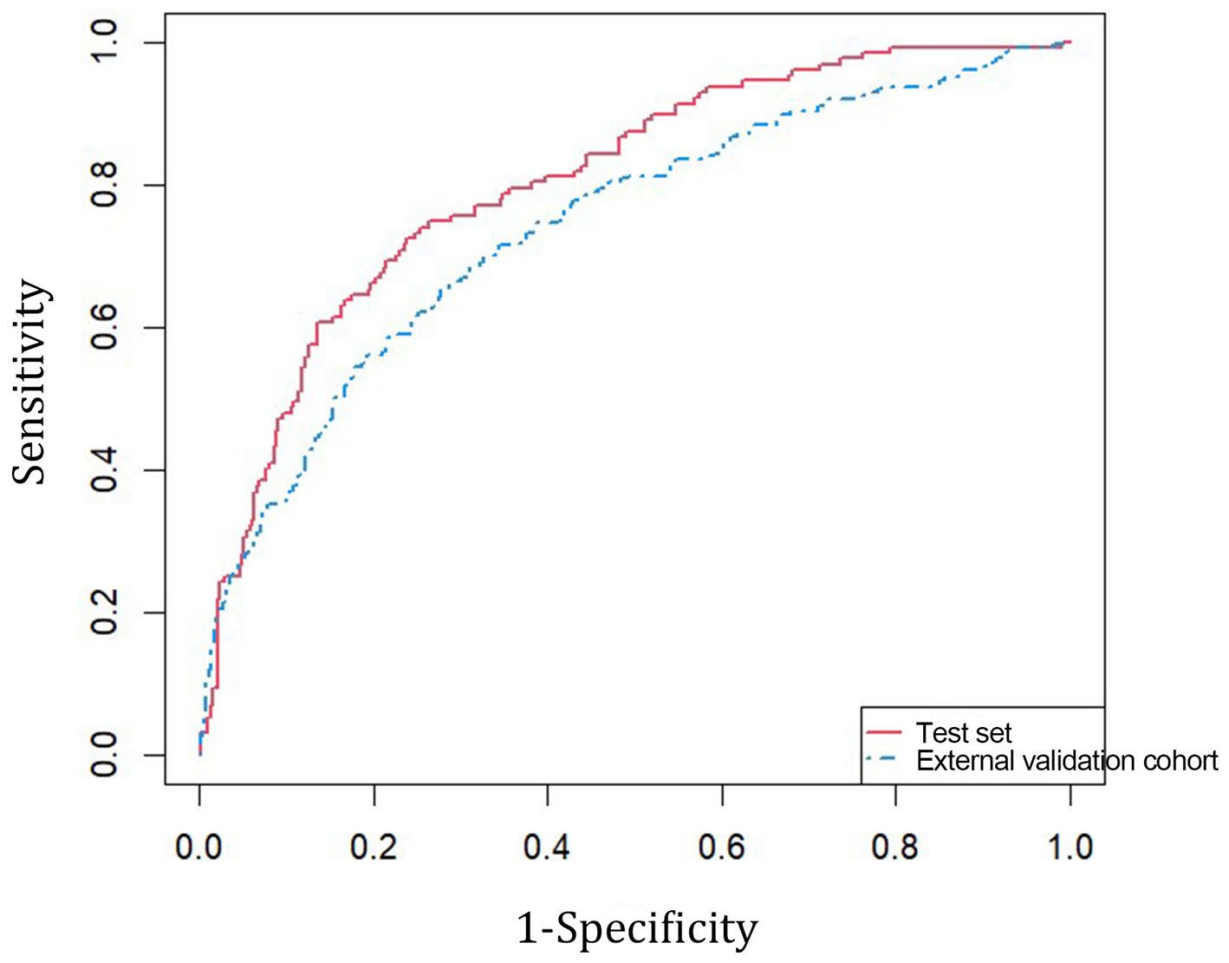
Internal and external validation ROC curves of Cox regression model with RCS.

The consistency index of the Cox regression model with RCS in the training set was 0.772. Validation of the discrimination ability of the model was performed using the AUC of the ROC curves. The AUC for internal validation was 0.803, which indicated that our model had good predictive discrimination in the test set. Likewise, the AUC for external validation was 0.740, indicating that the performance in the external cohort was worse than that in the internal cohort. However, the model had a relatively good predictive ability overall (Figure 4).

## Discussion

In the current study, we used the data from the recent three surveys of Chinese Longitudinal Healthy Longevity Survey (CLHLS) (2021) to include the most representative and comprehensive elderly illiterate Chinese women. Unlike previous studies, we provided a specific tool for those elderly illiterate women to predict the risk of developing cognitive impairment. Age, IADL and ADL score, WHtR, baseline MMSE, psychological score, and tooth brushing frequency were identified as the most important influencing factors for cognitive impairment in this population.

Specifically, the results of our study showed that age was the most important predictor for cognitive impairment in elderly illiterate women. It was also found that the relationship between age and the risk of cognitive impairment was nonlinear, which was in consistency with previous studies (Petersen et al., 2001; Lydon et al., 2022). Zhang et al. (2020) found that the effect of age on cognitive impairment increased rapidly among those 80 to 100 years old, which was also supported by our results, as the risk of cognitive impairment was significantly higher in the elderly illiterate females≥83 years with the rate increased year by year. Such observation pointed out that the risk of developing cognitive impairment increases considerably at an advanced age.

There were evidences indicating that functional disability (ADL and IADL) was strongly associated with the decline of cognition (Kuo et al., 2007; Carlo et al., 2016). Several studies (Kuo et al., 2007; Zhou et al., 2020) had put ADL and IADL in an equally important position. But in our study, IADL was highly correlated with the risk of developing cognitive impairment in elderly illiterate women, while ADL was weakly associated with cognitive impairment. A Korean cohort study (Lyu and Young, 2016) suggested that IADL limitations (OR = 1.15, 95% CI = 1.03–1.28) was a significant predictor of cognitive impairment only among women, whereas ADL was not. This result was similar to our study, which suggested that there was a tendency for IADL to be a stronger predictor compared with ADL for women. The possible explanation was that some IADL functions (housework, shopping, and childcare) were almost daily routines for women, thus the limitation on these functions may have a worse impact on cognitive function for women.

Our study suggested that the lower the baseline psychological score was, the higher the risk of future cognitive impairment became. And this finding was consistent with the results of previous studies. Paul et al. (2010) investigated the cognitive status of 1,286 adults aged ≥50 in Portugal, and they found that people with psychological distress were 2.6 times more likely to report cognitive impairment than those without psychological distress. Moreover, a previous study (Wu et al., 2016) concluded that agedness and low educational level had negative effects on psychological resilience. This indicated that we should pay more attention to the low-educated elderly adults with mental illness.

BMI was reported more in relevant studies compared with WHtR. However, the influence of BMI on cognition was inconclusive (Qu et al., 2020). Some studies showed that overweight and obesity were detrimental to cognitive function (Albanese et al., 2017); while some studies suggested that overweight was a protective factor (Nancy and Mary, 2009; Tao et al., 2018); and Giudice et al. (2016) discovered lower BMI was associated with cognitive impairment. Thus, Lyu and Young (2016) suggested adding WHtR or waist circumference to explain the effect of weight on cognitive impairment. In our study, we found that higher WHtR reduced the risk of cognitive impairment in elderly illiterate women. This might be related to the effect of body fat on cognitive function in old age. Older adults with more adipocytes tended to have higher estrogen levels and estrogen could protect their cognitive function (Sgarcia et al., 2014).

In addition, participants who brushed twice a day or more had a 41.4% lower risk of cognitive impairment compared to those who never brushed. Olsen and Singhrao (2020) suggested that inadequate oral hygiene may result in oral dysbiosis as a plausible contributory factor in the development of certain neurological diseases like Alzheimer’s disease. Poole et al. (2013) also reported that species from the oral microbiome such as *porphyromonas gingivalis* can be found in the brain. This destabilized the immune balance and can lead to the development of dementia. Some studies have shown that the average frequency of toothbrushing per day was lower in those with a low education level (YuRin and HyunKyung, 2021). The increased concern for their own health correlated to a higher level of awareness and attitudes about oral health care (Sapuric and Tozja, 2015). Maintaining good oral hygiene habits such as brushing twice a day and timely plaque-cleaning can help reduce the risk of developing cognitive impairment.

This study has several strengths. Firstly, our study was based on one of the largest national samples among elderly Chinese adults, which made the results more representative and convincing. Secondly, previous studies have analyzed older adults more as a whole. This study can identify the individuals at risk for cognitive impairment 3 years in advance among illiterate older women. These women are more likely to have cognitive impairment compared to the general elderly. Thirdly, the variables identified by our model were easily obtained and can reflect the common status of elderly illiterate Chinese women. Lastly, compared with other models (Zhou et al., 2020; Hu et al., 2022), as age was not satisfied with the PH assumption, the RCS Cox regression model we chose was better applicable than the traditional Cox model.

The limitations of this study should be acknowledged. Firstly, a selection bias might be observed due to excluding participants who died or failed to complete the survey from the present analysis. Secondly, the cognitive function was measured in 3-year intervals, and it was impossible to determine the specific time when cognitive impairment occurred, which may result in information bias. Thirdly, although we tried to include as many variables as possible, there was still the possibility some key factors could be missed during the process.

In the future, researchers can try to dig up some newer and more comprehensive databases and take into account information about the lost and deceased. Besides, we should pay more attention to the utilization and usefulness of predictive models in clinical practice. It is recommended to analyze biological and social factors together and take full advantage of external validation data.

## Conclusion

Age, MMSE score, WHtR, psychological score, ADL, IADL, and frequency of tooth brushing were shown in this study to be significantly associated with cognitive impairment. A nomogram was developed with good predictive performance and was used to predict 2-, and 3-year cognitive impairment risk for elderly illiterate Chinese women.

## Data availability statement

The original contributions presented in the study are included in the article/Supplementary material, further inquiries can be directed to the corresponding authors.

## Ethics statement

The use of CLHLS data was approved by the Biomedical Ethics Committee of Peking University.

## Author contributions

ZC designed the study and analyzed and interpreted the data. JD contributed to writing of the draft and critical revision of the manuscript. All authors contributed to the article and approved the submitted version.

## Funding

This study was supported in part by grants from the National Natural Science Foundation of China (Nos. 31971138 and 32270186).

## Conflict of interest

The authors declare that the research was conducted in the absence of any commercial or financial relationships that could be construed as a potential conflict of interest.

## Publisher’s note

All claims expressed in this article are solely those of the authors and do not necessarily represent those of their affiliated organizations, or those of the publisher, the editors and the reviewers. Any product that may be evaluated in this article, or claim that may be made by its manufacturer, is not guaranteed or endorsed by the publisher.
