# Supplementary material for: A prediction model of cognitive impairment risk in elderly illiterate Chinese women
Source: Front Aging Neurosci. 2023 Apr 26;15:1148071. doi: 10.3389/fnagi.2023.1148071 (PMC10169753; doi:10.3389/fnagi.2023.1148071)
Supplement: Supplementary file 1 [file Data_Sheet_1.docx]

**SUPPLEMENTARY MATERIAL**

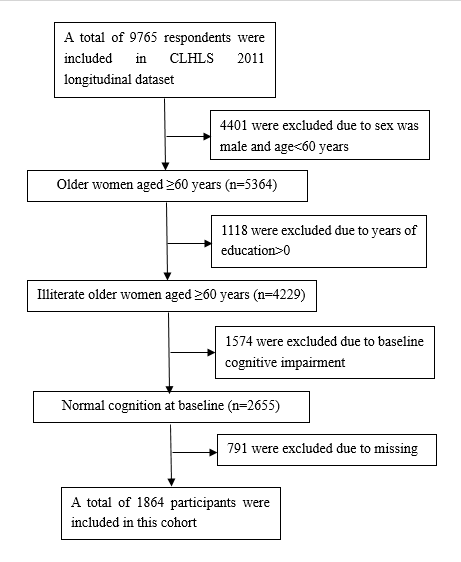


Supplementary Figure 1-A. Flow chart of participant selection in the development cohort (2011-2014).

**
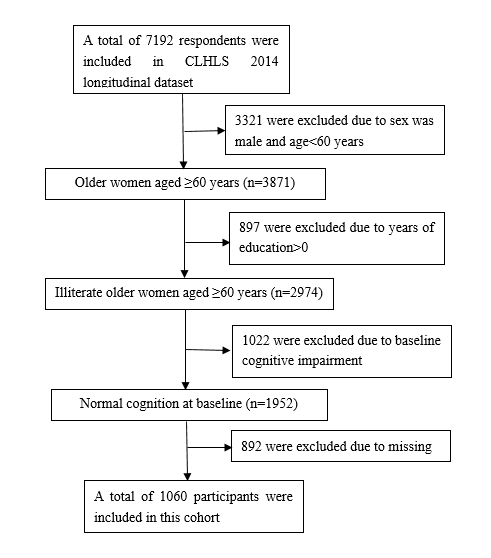
**

Supplementary Figure 1-B. Flow chart of participant selection in the validation cohort (2014-2018).

Supplementary Figure 2. Research Technology Route.

The project of “Chinese Longitudinal Healthy Longevity Survey” in China

Survey data 2011-2014, 2014-2018

Random Survival Forest Variable Primary Screening

Cleaning up the variables and calculating the outcome variables

Constructing RSF-Cox models from 2011-2014 CLHLS cohort data

Internal validation

External validation of CLHLS 2014-2018

AUC

Model Adjustment

Model Optimal

Output optimal model

Paper Writing

No

Yes

Supplementary Table 1. MD and VIMP values of the selected variables for the initial screening random survival forest model.

| Variables | | MD | VIMP value | Variables | MD | VIMP value |
| --- | --- | --- | --- | --- | --- | --- |
| Age | | 1.66 | 0.262 | Frequency of newspaper reading | 11.03 | 0.004 |
| IADL Score | | 3.94 | 0.044 | Sleep time | 11.04 | 0.001 |
| MMSE Score | | 5.51 | 0.022 | Staple food serving size | 11.17 | 0.002 |
| Psychological score | | 7.24 | 0.009 | Blood pressure type | 11.44 | 0.003 |
| Staple food serving size | 7.61 | | 0.012 | The first person to talk to when you have something on your mind | 11.71 | 0.003 |
| Types of cooking oil | | 7.97 | 0.023 | Alimony paid by the son | 11.81 | 0.001 |
| Number of Teeth | | 8.00 | 0.005 | The ability to touch the back of the hand | 11.82 | 0.005 |
| WHtR | | 8.19 | 0.005 | Total amount of money given to grandson in the previous year | 11.89 | 0.003 |
| Frequency of tooth brushing | | 8.25 | 0.018 | Alimony paid by grandson | 11.90 | 0.003 |
| ADL Score | | 8.34 | 0.012 | Housing Purchase | 11.92 | 0.003 |
| Frequency of doing housework | | 8.81 | 0.009 | Housing Type | 12.32 | 0.001 |
| Nearest Medical Distance | | 8.95 | 0.004 | Daughter's alimony payments | 12.46 | 0.001 |
| Number of children | | 9.60 | 0.004 | The ability to lift the arm up | 12.47 | 0.003 |
| Daily chat objects | | 9.62 | 0.005 | Ability to use chopsticks | 12.54 | 0.006 |
| Number of sons | | 10.01 | 0.006 | Types of fuel for daily cooking | 12.57 | 0.004 |
| Province of current residence | | 10.07 | 0.005 | Frequency of watching TV | 12.65 | 0.003 |
| BMI | | 10.16 | 0.007 | Housing ownership status | 12.68 | 0.002 |
| Frequency of eating fresh vegetables | | 10.33 | 0.011 | Listening | 12.80 | 0.010 |
| Number of trips in the previous year | | 10.34 | 0.010 | Like neat and clean | 12.81 | 0.006 |
| Daily diet taste | | 10.58 | 0.004 | Household Expenditure Determiner | 12.82 | 0.002 |
| Frequency of poultry rearing | | 10.70 | 0.009 | You are in charge of your own affairs | 12.86 | 0.003 |
| Province of birth | | 10.84 | 0.004 | Caregivers in sickness | 12.87 | 0.001 |
| The first person to turn to in case of trouble | | 10.99 | 0.006 | The ability to touch the heel of the neck with the hand | 13.13 | 0.004 |


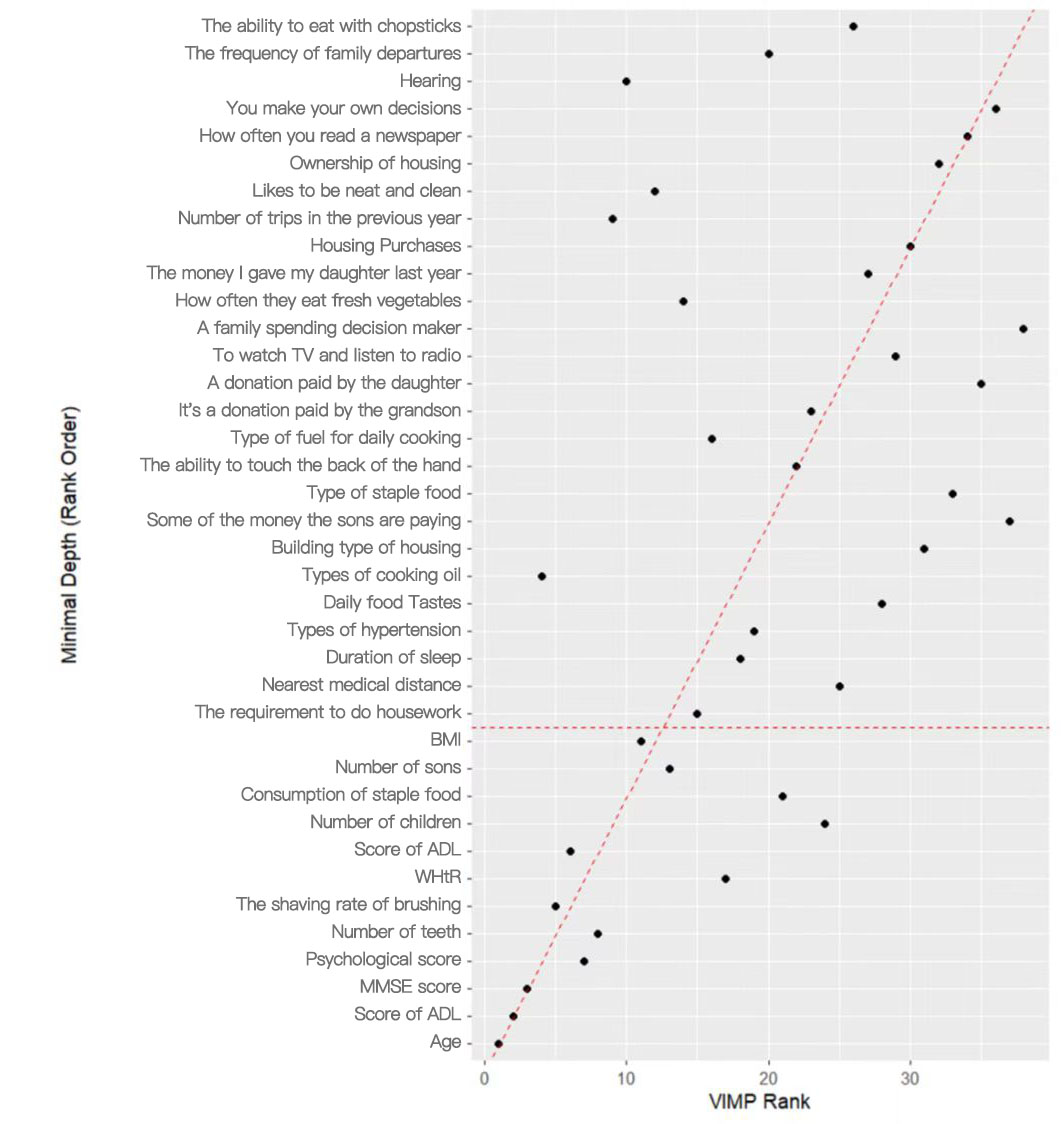


Supplementary Figure 3. Scatter plot of MD and VIMP methods for random survival forest mode.

Note: The points on the red dashed diagonal line represented the same ranking of the variables calculated by the MD and VIMP methods.

Supplementary Table 2. Univariate Cox regression analysis of influences related to cognitive impairment.

|  | Single factor analysis | | | | | |  | | PH test | |
| --- | --- | --- | --- | --- | --- | --- | --- | --- | --- | --- |
| Variables | *β* | Wald | S.E. | *P* | *HR* (95% *CI*) | |  | | *χ^2^* | *P* |
| Age | 0.08 | 219.00 | 0.01 | <0.001 | 1.08 (1.07-1.09) |  | | 25.60 | | <0.001 |
| IADL Score | 0.10 | 119.00 | 0.02 | <0.001 | 1.11 (1.09-1.13) | |  | | 4.36 | 0.037 |
| MMSE Score | −0.11 | 50.60 | 0.71 | <0.001 | 0.90 (0.87-0.93) | |  | | 1.29 | 0.256 |
| WHtR | −2.68 | 14.30 | 0.02 | <0.001 | 0.07 (0.02-0.28) | |  | | 1.84 | 0.175 |
| Psychological score | −0.04 | 6.06 | 0.01 | 0.014 | 0.96 (0.94-0.99) | |  | | 0.11 | 0.737 |
| BMI | −0.04 | 9.07 | 0.01 | 0.003 | 0.96 (0.93-0.99) | |  | | 0.84 | 0.360 |
| Number of Teeth | −0.04 | 31.20 | 0.02 | <0.001 | 0.96 (0.95-0.97) | |  | | 3.07 | 0.080 |
| Staple food eating | −0.08 | 9.38 | 0.03 | 0.002 | 0.93 (0.88-0.97) | |  | | 2.06 | 0.151 |
| ADL Score | 0.19 | 52.20 | 0.03 | <0.001 | 1.21 (1.15-1.27) | |  | | 0.51 | 0.476 |
| Number of Children | −0.03 | 1.50 | 0.04 | 0.221 | 0.97 (0.92-1.02) | |  | | 0.75 | 0.385 |
| Number of Sons | −0.07 | 2.97 | 0.01 | 0.085 | 0.94 (0.87-1.01) | |  | | 1.58 | 0.208 |
| Brushing frequency |  | 59.60 |  | <0.001 |  | |  | | 8.46 | 0.037 |
| No brushing |  |  |  |  | Reference | |  | |  |  |
| Occasional brushing | −0.23 |  | 0.19 | 0.216 | 0.79 (0.55-1.15) | |  | |  |  |
| Once a day | −0.85 |  | 0.14 | <0.001 | 0.43 (0.33-0.56) | |  | |  |  |
| Twice a day or more | −1.34 |  | 0.25 | <0.001 | 0.26 (0.16-0.42) | |  | |  |  |
